# Comparative Gut Microbiome in *Trachypithecus leucocephalus* and Other Primates in Guangxi, China, Based on Metagenome Sequencing

**DOI:** 10.3389/fcimb.2022.872841

**Published:** 2022-05-04

**Authors:** Tengcheng Que, Xianwu Pang, Hongli Huang, Panyu Chen, Yinfeng Wei, Yiming Hua, Hongjun Liao, Jianbao Wu, Shousheng Li, Aiqiong Wu, Meihong He, Xiangdong Ruan, Yanling Hu

**Affiliations:** ^1^ Terrestrial Wildlife Rescue and Epidemic Diseases Surveillance Center of Guangxi, Nanning, China; ^2^ Collaborative Innovation Centre of Regenerative Medicine and Medical BioResource Development and Application, Guangxi Medical University, Nanning, China; ^3^ Guangxi Center for Disease Control and Prevention, Nanning, China; ^4^ The First Affiliated Hospital of Guangxi Medical University, Nanning, Guangxi, China; ^5^ Life Sciences Institute, Guangxi Medical University, Nanning, China; ^6^ School of Information and Management, Guangxi Medical University, Nanning, China; ^7^ Acdemy of Inventory and Planning, National Forestry and Grassland Administration, Beijing, China; ^8^ Center for Genomic and Personalized Medicine, Guangxi key Laboratory for Genomic and Personalized Medicine, Guangxi Collaborative Innovation Center for Genomic and Personalized Medicine, Guangxi Medical University, Nanning, China

**Keywords:** *Trachypithecus leucocephalus*, gut microbiome, dietary, karst limestone, metagenomic sequencing

## Abstract

The *Trachypithecus leucocephalus* (white-headed langur) is a highly endangered, karst-endemic primate species, inhabiting the karst limestone forest in Guangxi, Southwest China. How white-headed langurs adapted to karst limestone and special dietary remains unclear. It is the first time to study the correlation between the gut microbiome of primates and special dietary, and environment in Guangxi. In the study, 150 fecal samples are collected from nine primates in Guangxi, China. Metagenomic sequencing is used to analyze and compare the gut microbiome composition and diversity between white-headed langurs and other primates. Our results indicate that white-headed langurs has a higher diversity of microbiome than other primates, and the key microbiome are phylum Firmicutes, class Clostridia, family Lachnospiraceae, and genera *Clostridiates* and *Ruminococcus*, which are related to the digestion and degradation of cellulose. Ten genera are significantly more abundant in white-headed langurs and François’ langur than in other primates, most of which are high-temperature microbiome. Functional analysis reveals that energy synthesis-related pathways and sugar metabolism-related pathways are less abundant in white-headed langurs and François’ langur than in other primates. This phenomenon could be an adaptation mechanism of leaf-eating primates to low-energy diet. The gut microbiome of white-headed langurs is related to diet and karst limestone environment. This study could serve as a reference to design conservation breeding, manage conservation units, and determine conservation priorities.

## Introduction

The white-headed langur is a highly endangered, karst-endemic primate species with a current wild population of only 1,100 individuals (Konstant et al., 2003). The species inhabits the karst limestone forest in Guangxi, Southwest China, and belongs to the national I class key protected animals (Huang, 2002). White-headed langurs mainly feed on woody plants, which especially focusing on several main plants. Thus, white-headed langurs feed on the tender leaves and buds in the treetops to drink water (Li et al., 2003; Li and Rogers, 2006). In the dry season, white-headed langurs can only feed on old leaves, which contain much less water than new ones. Thus, white-headed langurs adapted to their seasonal habitats by adjusting their ranging behavior and spent more time resting and less time moving and feeding (Huang, 2002; Qihai et al., 2013).

The composition of the gut microbiome was influenced by several factors, including maternal delivery, genetic, geography, and lifestyle (Khan et al., 2016). Diet was an important factor determining the composition of the gut microbiome. Dietary fiber can regulate the contents of Firmicutes and Bacteroidetes, the two main phyla of the human gut microbiome, and increased the abundance of probiotics, such as *Lactobacillus*, *Bifidobacterium*, and cellulolytic bacteria (Canfora et al., 2019; Coker et al., 2021). Bacterial abundance was also modulated by dietary macronutrient consumption, including proteins, carbohydrates, and fats (Hildebrandt et al., 2009; Turnbaugh et al., 2009). A previous study found differences in the gut microbiome between captive and wild primates, indicating that captivity humanizes the primate microbiome (Clayton et al., 2016). These data suggest that diet influences the gut microbiome. White-headed langurs mainly feed on leaves, which are coarse fiber and difficult to digest. Whether or not the gut microbiome of white-headed langurs is influenced by their diet is unclear. Over time, the stomachs of white-headed langurs have changed to form a cavity that allows cellulose-breaking bacteria to survive (Zhou et al., 2011). We inferred that the gut microbiome in white-headed langurs is related to their diet.

The karst limestone where white-headed langurs habitated was a harsh environment containing various caves (Huang and Li, 2005). Caves were typical features of a subsurface karst, characterized by darkness, low-to-moderate temperatures, high humidity, and limited nutrients (Gabriel and Northup, 2013). Despite their oligotrophic conditions, microbial communities thrived in caves, with the average number of microorganisms growing in these ecosystems reaching 10^6^ cells/g of rock (Barton and Jurado, 2007). Extremophiles can colonize extreme environments, and they were the sources of novel biomolecules and metabolic pathways (Rothschild and Mancinelli, 2001; Seufferheld et al., 2008). White-headed langurs lived in caves and drunk water in caves when they were thirsty (Huang and Li, 2005). However, whether or not the environment affects the gut microbiome of white-headed langurs was unclear.

In the present study, we collected fecal samples from nine primates, including white-headed langur, François’ langur, silvered langur, loris, pygmy loris, ring-tailed lemur, macaques, gibbon, and baboon, in Guangxi, China. We analyzed and compared the gut microbiome composition and diversity of white-headed langurs and other primates by using high-throughput sequencing. We predicted the functional pathways of the gut microbiome and explored the correlation between environment, diet, and the gut microbiome in white-headed langurs. This study could help improve and protect the living environment of white-headed langurs.

## Materials and Methods

### Subjects and Sample Collection

One-hundred fifty fecal samples were collected from white-headed langur (27), François’ langur (20), silvered langur (9), loris (19), pygmy lorises (8), ring-tailed lemur (23), macaques (34), gibbon (5), and baboon (5) in Guangxi Land Wildlife Rescue Research and Epidemological Surveillance Center, Nanning Zoo, Chongzuo White-Headed Langur Nature Reserve, Gupu Mountain Nature Reserve in Hezhou, and Wuzhou Langur Breeding Center, all of them were adult. All samples were immediately scooped out with a sampling spoon. Fresh feces that were not contaminated in the middle of the feces were placed into a sample tube and then placed in a box containing dry ice. These samples were immediately transported to the laboratory for storage under −80°C. The whole process abided by the natural wildlife protection law and did not produce any harmful substances to the environment and animals. All samples were collected from March to May in 2018.

### DNA Extraction and Metagenomic Sequencing

Microbial DNA was extracted from 200 mg of frozen fecal samples using the Qiagen DNA extraction kit (Qiagen, Germany) in accordance with the manufacturer’s kit protocols. DNA libraries were prepared using the TruSeq DNA Sample Preparation Guide (Illumina, 15026486 Rev.C). Library quantity was assessed with a Qubit2.0 (Thermo Fisher Scientific, USA). Libraries were then sequenced on an Illumina HiSeq X-ten platform (Illumina, USA) to generate 150-bp paired-end reads.

### Data Pre-Processing

Unassembled sequencing reads were preprocessed by trimming 1) reads that overlap with the adapter over a certain threshold (5 bp); 2) low-quality bases (Q-value ≤ 19, and accounts for more than 50% of the total base); and 3) reads that contain N nucleotides over a certain threshold (≥5%). Clean data of all samples were processed using FastQC (Wingett and Andrews, 2018) for quality control and MultiQC (Ewels et al., 2016) for integration. After these two steps, clean data with high quality were obtained.

### Analysis of Whole Metagenome Sequencing Data

We used the Kraken2 program to analysis microbiome using the default parameters (Wood et al., 2019). The clean paired-end sequence reads were used for Kraken2 analysis against the PlusPFP database containing archaea, bacteria, viral, human and UniVec_core. The relative abundance of species from the Kraken2 analysis was calculated using the MetaPhlAn2 (Truong et al., 2015) and ChocoPhlAn pan-genomic databases (Franzosa et al., 2018) with default settings. These reads were then used for functional profiling using HUMAnN2 (Abubucker et al., 2012) to estimate the relative abundance of microbial gene and MetaCyC pathways using the Uniref50 database with the default settings. Differentially abundant species were identified using MaAsLin. For both gene families and metabolic pathways, α-diversity was evaluated by Shannon index, and β-diversity was evaluated by Bray–Curtis distance (Liu et al., 2021). Using the generated taxonomic abundance and function abundance tables, we performed Principal Coordinates Analysis (PCoA), linear discriminant analysis (LDA), diversity index, and richness index analyses. Grouping information generated from the above analyses was used for LDA effect size (LEfSe) multivariate statistical analysis and comparative analysis of metabolic pathways to explore species composition and functional differences between different groups.

### Statistical Analysis

Stamp (Parks et al., 2014) was used to conduct a visual analysis of similarity or difference through abundance. The results of microbiome composition, taxonomic level, and relative abundance are presented in the form of a column chart. The relative abundance at the phylum and genus levels was mapped by an online website (http://www.ehbio.com/ImageGP/Ind-ex.php/Home/Index/index.html) and origin (2017) software. LEfSe software (Segata et al., 2011) was used to compare the groups with significant difference in abundance from phylum to genus. LDA was used to estimate the influence of abundance of each species on the difference effect. R software was used to calculate the diversity index (Shannon index and Simpson index) and richness index (Chao index and ACE index), which are presented in the form of a box diagram. Unpaired t-test was used to compare the metabolic pathways between different groups using GraphPad Prism 7.

## Results

### Microbiome Diversity Analyses

**Figure 1:**
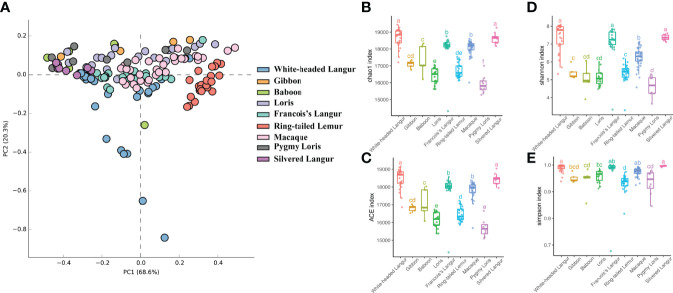
PCoA of the gut mirobiome and boxplots of the diversity index and richness index. **(A)** PCoA of structure differentiation and interindividual similarity on the gut microbiota of nine primates. **(B)** Boxplots of the Chao1 index. **(C)** Boxplots of the ACE index. **(D)** Boxplots of the Shannon index. **(E)** Boxplots of the Simpson index.

PCoA based on the Bray–Curtis distance revealed the dissimilarity of bacterial communities among species. In this study, the phylum level of the microbiome was analyzed by PCoA. The closer the points are, the higher the similarity is. Results showed that the distribution of the microbiome in white-headed langur was dispersed, whereas that in other species were clustered, especially the gut microbiome in ring-tailed lemur (**Figure 1A**). The Chao1 and ACE indexes showed that white-headed langur and silvered langur had the highest index, followed by François’ langur and macaque, and the lowest was loris and pygmy lorises, indicating that the diversity of the microbiome in white-headed langur and silvered langur was the highest (**Figures 1B, C**). The Shannon and Simpson indexes also showed that white-headed langur and silvered langur had the highest index, followed by macaque, and the lowest was pygmy lorises (**Figures 1D, E**). No significant difference was found between white-headed langur and silvered langur.

### Abundance Analysis and Taxonomy Annotation

**Figure 2:**
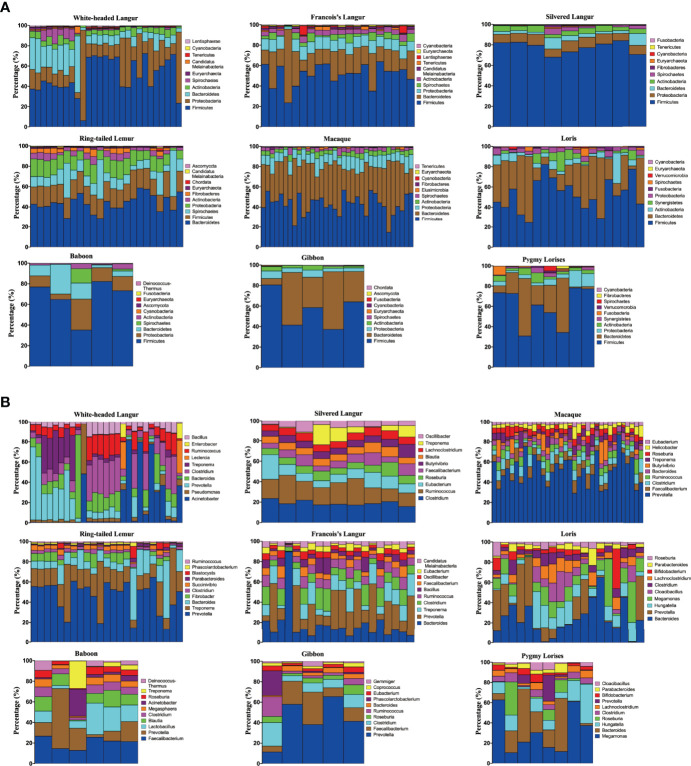
Taxonomy annotation. **(A)** Taxonomy annotation in phylum. **(B)** Taxonomy annotation in genus.

The microbiomes in white-headed langur and other primates were annotated using Kraken2 and HUMAnN2 software, respectively. In the study, 65 bacterial phyla were annotated in all 150 samples by using Kraken2. The top 10 phyla are presented in **Figure 2A**. Sixty-four phyla were annotated in white-headed langur. The most abundant phylum microbiome was Firmicutes, followed by Proteobacteria, Bacteroidetes, and Actinobacteria. The same to other omnivorous primates. At the genus level, 2,769 were annotated in white-headed langur, and the top 10 genera were presented in **Figure 2B**. The most abundant genus bacteria in white-headed langur were *Acinetobacter*, *Pseudomonas*, *Prevotella*, *Bacteroides*, *Clostridium*, *Leclercia*, *Treponema*, *Ruminococcus*, *Enterobacter*, and *Bacillus*. The major genus bacteria of the nine primates differed from one another. The microbiome was also annotated using HUMAnN2 software.

### Differences in Gut Microbiome Composition

**Figure 3:**
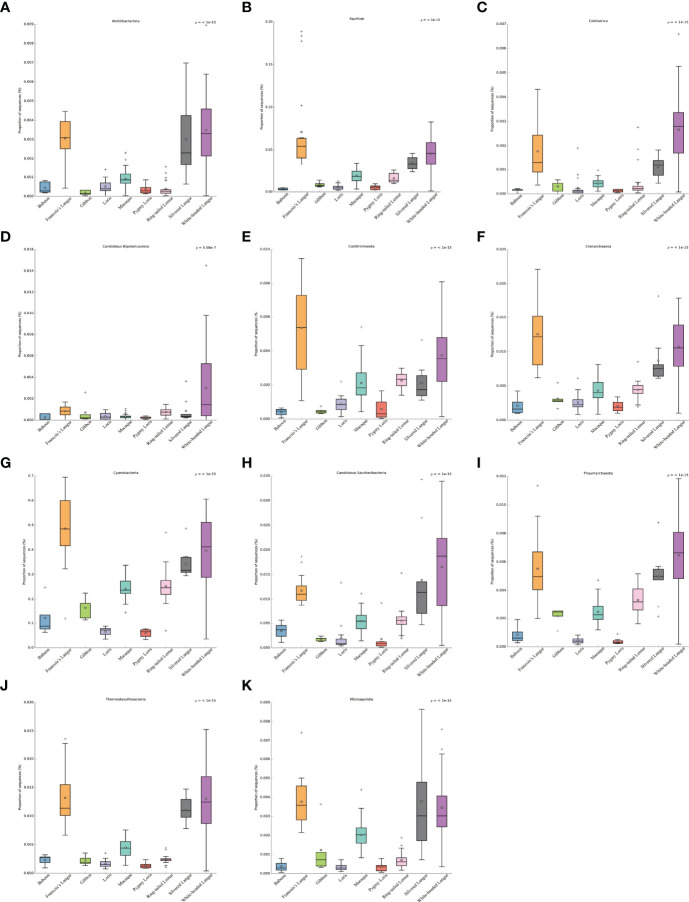
Comparison of gut microbial composition between white-headed langur and other primates in phylum. **(A–K)** Abditibacteriota, Aquificae, Caldiserica, Candidatus Bipolaricaulota, Calditrichaeota, Crenarchaeota, Cyanobacteria, Candidatus Saccharibacteria, Thaumarchaeota, Thermodesulfobacteria, and Microsporidia are presented in the form of box diagram separately.

The comparison of microbial composition between white-headed langur and other primates at the phylum level was analyzed using Kruskal–Wallis sum rank test, and the *p*-value was adjusted by Benjamini–Hochberg. The following 10 phylum microbiome were more enriched in white-headed langur than in the other primates (*P ≤* 0.01): Abditibacteriota, Aquificae, Caldiserica, Candidatus Bipolaricaulota, Calditrichaeota, Crenarchaeota, Cyanobacteria, Candidatus Saccharibacteria, Thaumarchaeota, and Thermodesulfobacteria (**Figures 3A–J**). Candidatus Bipolaricaulota was highly abundant only in white-headed langur (**Figure 3D**). White-headed langur and François’ langur are leaf-eating primates and live karst limestone. Results showed that Abditibacteriota, Aquificae, Caldiserica, Calditrichaeota, Crenarchaeota, Cyanobacteria, Candidatus Saccharibacteria, Thaumarchaeota, and Thermodesulfobacteria were also more enriched in François’ langur. Microsporidia in white-headed langur, a specialized intracellular parasite, was associated with the death of patients with AIDS, transplantation, and immunocompromised diseases (**Figure 3K**).

### Biomarker Analyses of Gut Microbiome

**Figure 4:**
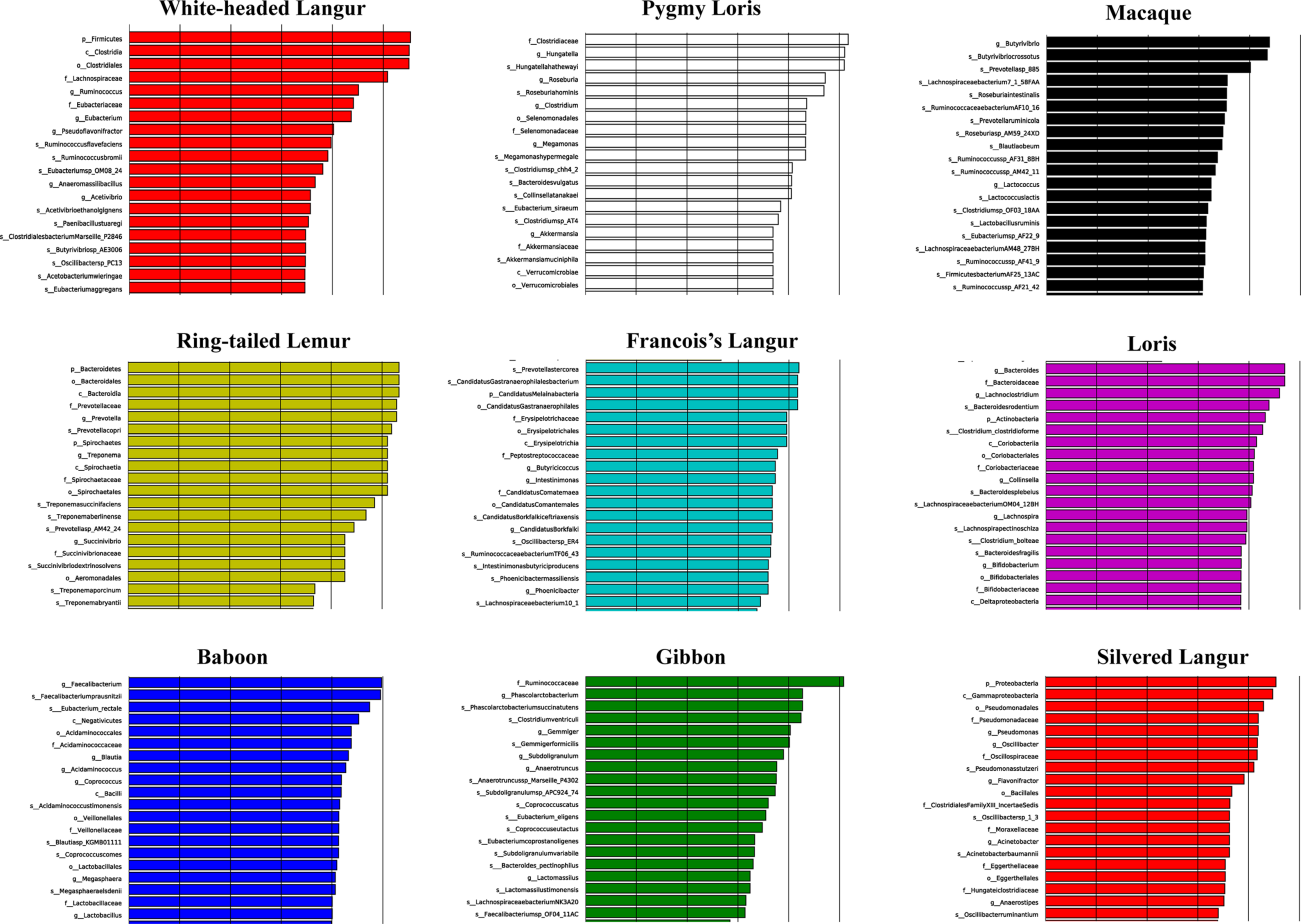
LDA value distribution histogram of the significantly different biomarkers in all primates. Top 20 are presented.

**Figure 5:**
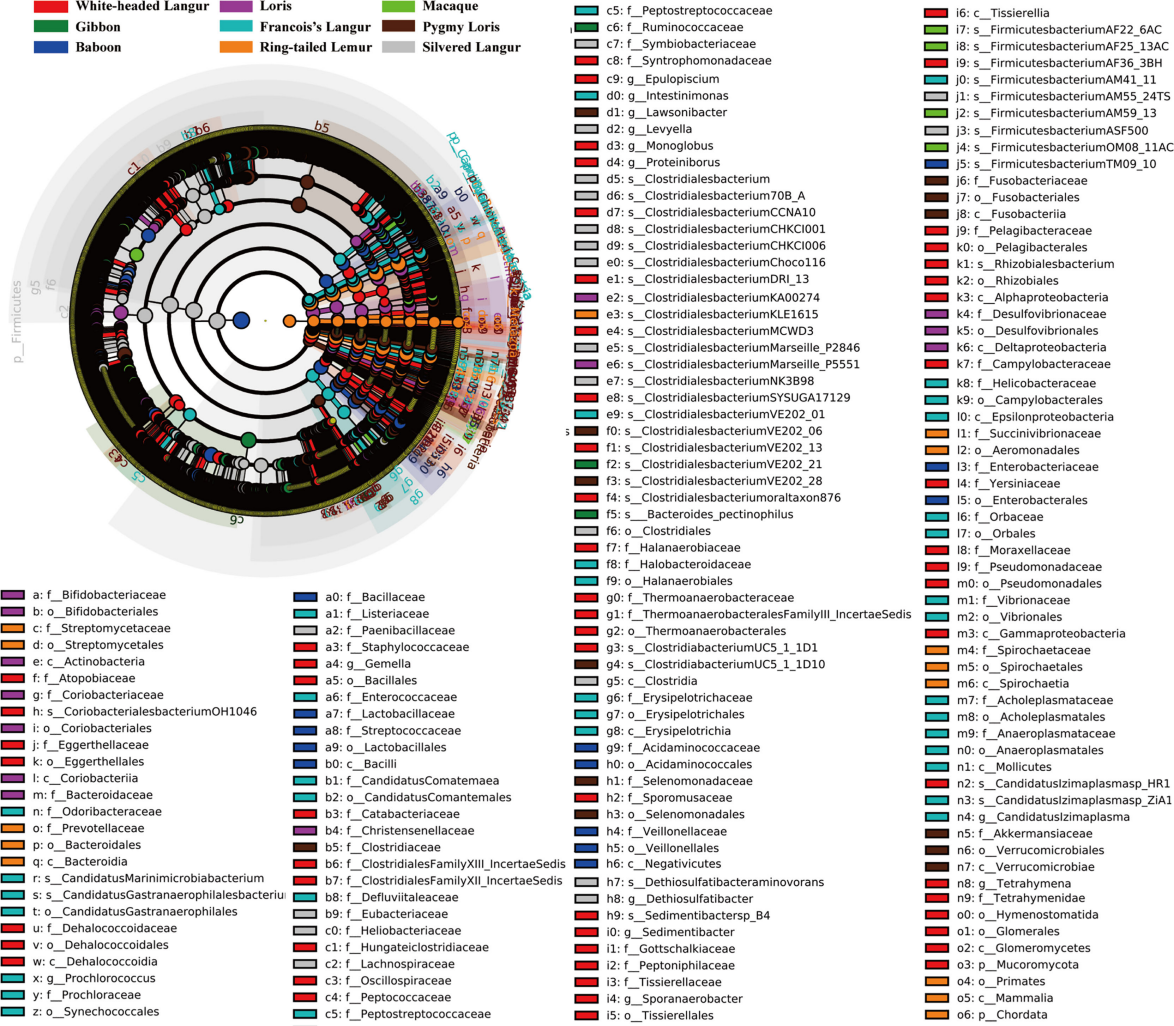
Cladogram of the significantly different biomarkers in all primates.

We used LEfSe to screen significantly different biomarkers in each group. LEfSe identifies genomic features (genes, pathways, or taxa), characterizing the differences between two or more biological conditions (or classes). Thus, this tool enables the characterization of microbial taxa specific to an experimental or environmental condition and identifies metagenomic biomarkers in different microbial communities. The LDA value distribution histogram (**Figure 4**) and cladogram (**Figure 5**) were used to present significantly different biomarkers. Results showed that the key microbiome in white-headed langur were phylum Firmicutes, class clostridia, family lachnospiraceae, and genera *Clostridiates* and *Ruminococcus*, which were related to the digestion and degradation of cellulose. The key microbiome in François’ langur were phylum Candidatus, class Melainabacteria, families Erysipelotrichaceae and Prevotellaceaegenus, and genus Gastranaerophilales. The key microbiome in silvered langur were phylum Proteobacteria, class Gammaproteobacteria, order Pseudomonadates, family Pseudomonadates, and genus *Pseudomonas*. The key microbiome of the nine primates differed from one another.

### Differences in the Functional Profiles of Gut Microbiome

**Figure 6:**
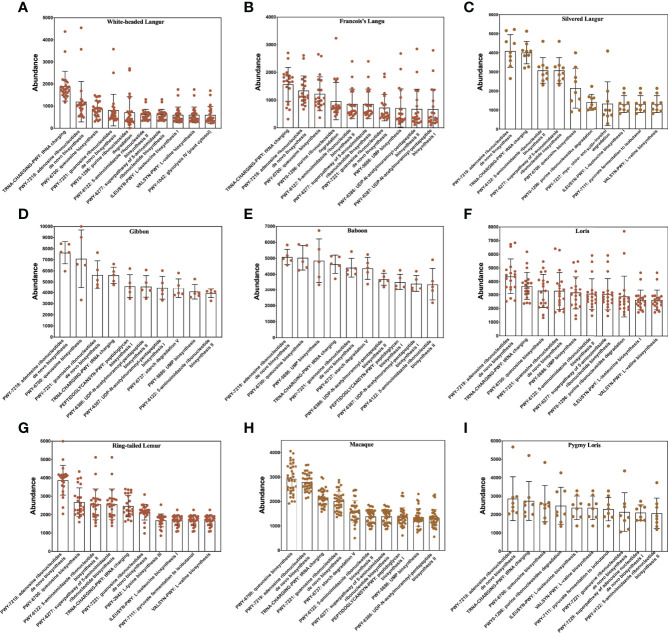
Top 10 functional pathways in all primates. **(A–I)** White-headed langur, François’ langur, silvered langur, gibbon, baboon, loris, ring-tailed lemur, macaque, and pygmy lorise, respectively.

**Figure 7:**
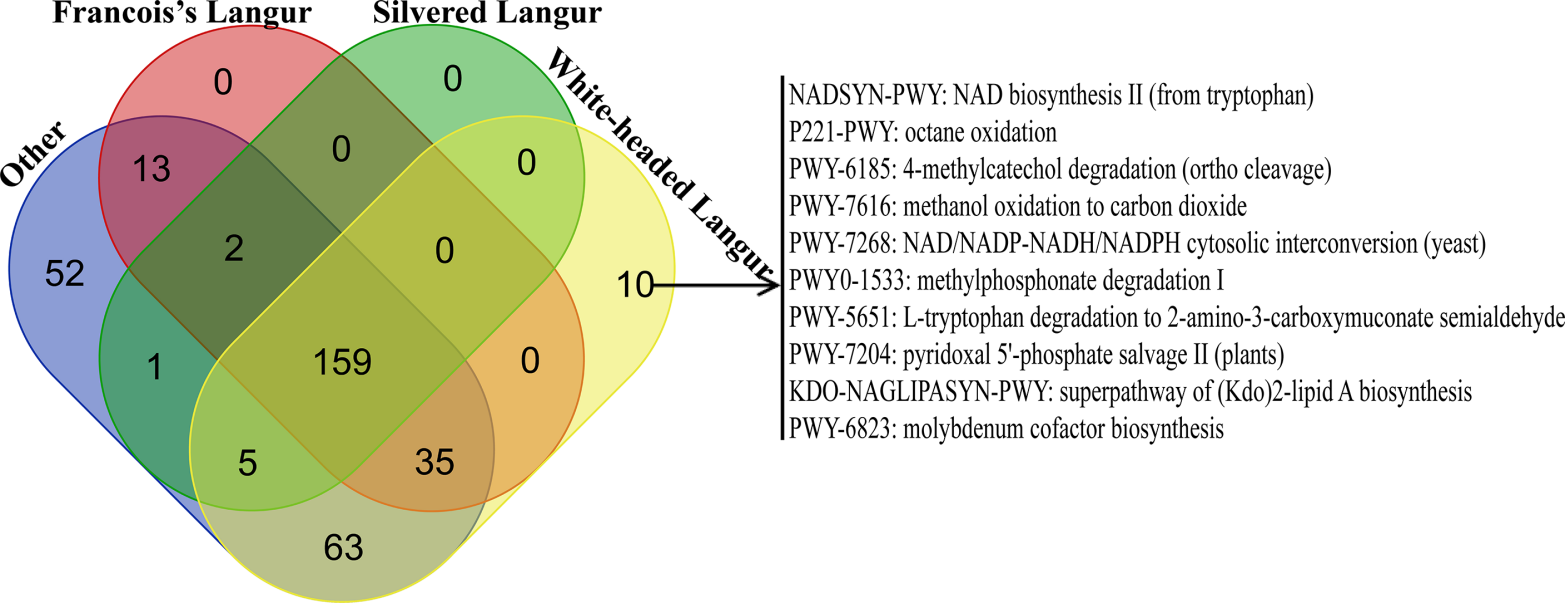
Venn diagram of functional pathway in all primates. Other included loris, pygmy lorises, ring-tailed lemur, macaques, gibbon, and baboon.

HUMAnN2 is effective for analyzing microbial functional pathway abundance with metagenomic and transcriptome data. This tool could analyze the microbial composition and functions using the MetaPhlAn2 and ChocoPhlAn databases. In this study, 274 functional pathways were annotated in white-headed langur in wild, whereas only 109 functional pathways were annotated in white-headed langur in captivity. However, only one white-headed langur in captivity was included in the study. The top 10 functional pathways in all primates are presented in **Figure 6**. Most of the top 10 functional pathways were about all types of ribonucleotide biosynthesis. However, the abundance of these functional pathways was lower in white-headed langur and François’ langur than in other primates, which may be related to diet and karst limestone. As shown in the Venn diagram in **Figure 7**, 10 functional pathways were unique to white-headed langur, including NADSYN-PWY (NAD biosynthesis II), P221-PWY (octane oxidation), and PWY-6185 (4-methylcatechol degradation) (**Figure 7**).

**Figure 8:**
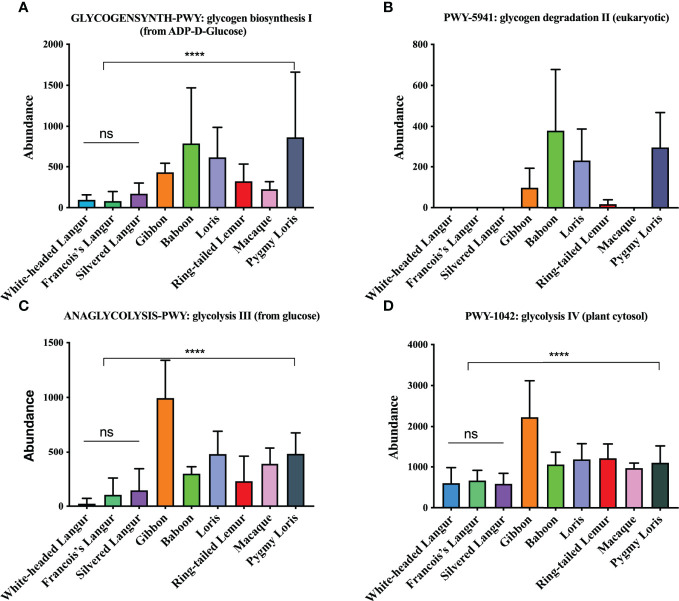
Metabolic pathways of the gut microbiome in all primates. **(A)** GLYCOGENSYNTH-PWY:glycogen biosynthesis I (from ADP-D-Glucose). **(B)** PWY-5941: glycogen degradation II (eukaryotic). **(C)** ANAGLYCOLYSIS-PWY:glycolysis III (from glucose). **(D)** PWY-1042:glycolysis IV (plant cytosol). ns represents no significant difference; **** represents *P* < 0.0001.

We also explored the metabolic pathways of the microbiome involved in glycolysis using Wilcoxon rank sum tests. The metabolic pathways included glycogen biosynthesis I, glycogen degradation II, glycolysis III, and glycolysis IV. White-headed langur, François’ langur, and silvered langur, all of which were leaf-eating primates, had the lowest abundance in the four metabolic pathways (**Figure 8**). No significant difference was found between the three leaf-eating primates (*P* > 0.05), whereas the other omnivorous primates had higher abundance than the leaf-eating primates (*P* < 0.0001), indicating that diet was associated with the metabolic pathways of the microbiome.

## Discussion

White-headed langurs inhabited the karst limestone forest in Guangxi, Southwest China, fed mainly on leaves, and spend 91.6% of their foraging time on leaves (Huang et al., 2008; Huang et al., 2017). However, how white-headed langurs adapt to the fibrous diet and the karst limestone remains unclear. In the study, result showed that the diversity of the microbiome was higher in white-headed langur than in other primates. The key microbiome in white-headed langur were phylum Firmicutes, class clostridia, family lachnospiraceae, and genera *Clostridiates* and *Ruminococcus*, which were related to the digestion and degradation of cellulose. In addition, 10 phylum microbiomes were more enriched in white-headed langur and François’ langur than in other primates, most of which were high-temperature microbiome. The relative abundance of functional pathways involved in glycogen synthesis and degradation in leaf-eating primates were extremely low, indicating the adaptation of white-headed langur to low-energy food.

Change in the gut microbiome was often strongly associated with diet (Barelli et al., 2015; Amato et al., 2016). Food consumption patterns, such as omnivore versus plant-based diet, were associated with changes in the human gut microbiome, which displayed increased abundance of *Ruminococcus* and *Streptococcus*, whereas those having a vegetable-based diet show increased abundance of *Roseburia*, *Lachnospira*, and *Prevotella* (De Filippis et al., 2016). Similar to that of humans, the microbiome of non-human primates contains Bacteroidetes, Firmicutes, and Proteobacteria (Yasuda et al., 2015; Nagpal et al., 2018). In our study, Firmicutes, clostridia, lachnospiraceae, *Clostridiates* and *Ruminococcus*, which were related to the digestion and degradation of cellulose. The gut microbiome enabled animals to absorb nutrients from the most complex polysaccharides, namely, cellulose and hemicellulose, because the animals themselves did not have the appropriate enzymes in their digestive system (Henderson et al., 2015; Li et al., 2015; Wang et al., 2019). Through degradation and fermentation, these microorganisms decompose plant cellulose into fatty acids and other nutrients to provide their daily energy requirement. Anaerobic and relatively aerobic bacteria (mainly Firmicutes and Bacteroidetes) were the most abundant, along with much smaller quantities of Proteobacteria, Fibrobacteres, Tenericutes, and Actinobacteria (Cholewinska et al., 2020). In the present study, the most abundant phylum in white-headed langur was Firmicutes, followed by Proteobacteria, Actinobacteria, and Bacteroidetes. LEfSe analysis also showed that Firmicutes was the dominant phylum in white-headed langur, and the dominant genera were *Clostridiates* and *Ruminococcus*, most of which are related to digestion and degradation. Firmicutes is almost a ubiquitous phylum in nature. Most of its members are spore-forming Gram-positive bacteria and are an essential part of the microbial community associated with plant cellulose degradation and carbohydrate polymer decomposition. Therefore, Firmicutes was important when ligninolytic bacteria and enzymes were desired (Liu et al., 2019). White-headed langurs mainly fed on and drink water from leaves, especially from tender leaves and buds (Huang and Li, 2005; Huang et al., 2008). However, these leaves are coarse cellulose and difficult to digest, and white-headed langurs depend on these bacteria to break down and digest these leaves. These bacteria may be indispensable for the host to adapt to extreme diets or environments. These bacteria were also found in François’ langur and silvered langur, indicating that the gut microbiome is associated with diet.

The microbial composition was mainly influenced by several factors, such as the environment, age, physiological state, diet, and even geographical differences (Henderson et al., 2015; Cholewinska et al., 2020). The karst limestone habitat of white-headed langurs was harsh; it features many cliffs and was covered with various caves (Li and Rogers, 2005). High-temperature limestone in karst is a hotbed of heat-loving bacteria, which could influence the gut microbiome of white-headed langur. In the present study, 10 phyla were significantly higher in white-headed langur than in the other primates, and most members of these phyla were high-temperature bacteria. For example, Abdibacteriota is a Gram-negative, oxygen-demanding, oligotrophic heterodoxygenic bacteria. It can grow under only a limited carbon source and can survive in low-nutrition environments. Phenotypic and genomic analyses have shown that Abdibacteriota was extremely resistant to antibiotics and toxic compounds (Tahon et al., 2018). Aquificae can reproduce in microalkaline sulfide hot springs at temperatures above 70°C and still have a strong nitrogen fixation capacity even under low concentrations of nitrogen compounds; they were considered the main producers of fixed carbon and organic compounds (Nishihara et al., 2018). Caldiserica was a chemical heterotrophic organism that reduced sulfur in hot springs and lived in environments with high temperatures, high salt concentrations, and high pressures, making it difficult to be separated and cultured in the laboratory (Martinez et al., 2019). Candidatus Bipolaricaulota only shown high abundance in white-headed langur. Candidatus Bipolaricaulota was mainly found in deep sea, lake sediments, hot springs, reservoirs, and salt-lake sediments, and it can break down sugar and proteins (Luiken et al., 2019). Calditrichaeota has been recently recognized a novel bacterial phylum with three cultured representatives, isolated from hydrothermal vents, and can degrade detrital proteins (Marshall et al., 2017). Candidatus Saccharibacteria was widely found in soil, sediment, wastewater, and animals, as well as in clinical settings; given its lack of 16S rRNA sequence, little was known about its biological function (Ferrari et al., 2014). Crenarchaeota had low diversity and is thought to be composed of extreme thermophilic bacterial, which was related to system development and metabolism. Thermodesulfobacteria, a small group of thermophilic sulfate-reducing bacteria (Merkel et al., 2017; Guo et al., 2020), can be found in hot springs at high temperatures (>70°C) and was highly abundant in white-headed langur. These high-temperature and cold-resistant bacteria probably originated in karst limestone surface and then transferred to the gut of the white-headed langur. The number of thermophilic bacteria in white-headed langur increased because of the impact of the environment, which helped them adapt to the limestone environment.

We aimed to identify the major function pathway of the gut microbiome in white-headed langurs. Results show that the overall abundance of functional pathways, especially the ribonucleotides biosynthesis related pathways, was lower in white-headed langurs and François’ langurs than in other primates. This result may be related to the energy synthesis because white-headed langur and François’ langur mainly feed on leaves and spend most of their time on rest. Similar to the sugar metabolism pathway, the abundance of sugar metabolism pathways was low in white-headed langur, François’ langur, and silvered langur but high in other omnivorous primates, such as loris, pygmy lorises, ring-tailed lemur, macaque, gibbon, and baboon. This result may be related to their diet because these leaves had less sugar than other food, which led to less sugar decomposition and glycogen synthesis. Thus, the hypometabolic pathway enables the adaptation of white-headed langurs to low-energy food. Ten biological functional pathways unique to white-headed langurs were also found. These pathways are mainly related to the oxidation and degradation of hazardous substances and the biosynthesis of substances. For example, adenine dinucleotide was involved in various physiological activities, such as cell substance metabolism, energy synthesis, and cell DNA repair, which played an important role in the body’s immunity. L-Tryptophan, one of the essential amino acids, was obtained from food. It is an important precursor for 5-serotonin, melatonin, canine uric acid, and niacin. These unique functional pathways could enhance the immunity of white-headed langurs and protect them from the damage of karst limestone.

## Conclusion

We analyzed and compared the gut microbiome composition and diversity of white-headed langurs and other primates and explored the influence of diet and karst limestone environment on the gut microbiome of white-headed langur. The gut microbiome in white-headed langur had a high diversity and was associated with diet and karst limestone. The unique functional pathways of white-headed langur were mainly related to the biosynthesis and degradation of harmful substances, enabling them adapting to the karst limestone environment. In addition, the abundance of sugar metabolism-related pathways was low, which was presumably an adaptation to low-energy diet. The results of this study suggest that the gut microbiome is influenced by different factors that help the host adapt to changes in diet and environment.

## Data Availability Statement

The datasets presented in this study can be found in online repositories. The name of the repository and accession number can be found at Genome Sequence Archive (Genomics, Proteomics, and Bioinformatics 2017) in BIG Data Center (Nucleic Acids Res 2019), Beijing Institute of Genomics (BIG), Chinese Academy of Sciences; https://bigd.big.ac.cn/gsa; CRA002281.

## Ethics Statement

The animal study was reviewed and approved by the Ethics Committee of the Guangxi Chongzuo White-Headed Langur National Nature Reserve Management Center.

## Author Contributions

TQ and YLH: conceived and designed the project. XP: wrote and edited the manuscript. HH, YW, and YMH: supported and performed laboratory experiments and sequencing. HH and XP: performed the genome assembly and bioinformatics analysis. XP and HH generated the figures and tables. PC, SL, AW, and MH: collected and prepared samples. HL, JW, XR, TQ and YH: review and editing. All authors contributed to the article and approved the submitted version.

## Funding

This study was funded by the Guangxi Key Research and Development Program (No: Guike AB20059002) and Innovation Project of Guangxi Graduate Education (No: YCBZ2021053). Department of forestry of Guangxi Zhuang Autonmous Region (Guilin Pre-Protection: 2016007).

## Conflict of Interest

The authors declare that the research was conducted in the absence of any commercial or financial relationships that could be construed as a potential conflict of interest.

## Publisher’s Note

All claims expressed in this article are solely those of the authors and do not necessarily represent those of their affiliated organizations, or those of the publisher, the editors and the reviewers. Any product that may be evaluated in this article, or claim that may be made by its manufacturer, is not guaranteed or endorsed by the publisher.
